# Spending with purpose: tracking health expenditures in Tajikistan to inform progress toward UHC^☆^

**DOI:** 10.1016/j.hpopen.2026.100164

**Published:** 2026-02-13

**Authors:** Baktygul Akkazieva, Ghafur Muhsinzoda, Malika Khakimova, Farrukh Egamov, Ilker Dastan, Bernd Rechel, Susannah Robinson, David Beran

**Affiliations:** a University of Geneva, Institute of Global Health, 9 Chemin des Mines, 1202 Geneva, Switzerland; b World Health Organization Country Office in Tajikistan, Dushanbe, 37/1 Bokhtar Street, Dushanbe 734019, Tajikistan; c Ministry of Health and Social Protection in Tajikistan, Dushanbe, 69 Shevchenko, Dushanbe 734000, Tajikistan; d European Observatory on Health Systems and Policies, London School of Hygiene & Tropical Medicine, London; e World Health Organization, Geneva, Switzerland; f University of Geneva, Division of Tropical and Humanitarian Medicine, Chemin Eugène-Rigot 2, 1202 Geneva, Switzerland

**Keywords:** Universal Health Coverage, Health financing reforms, Out of pocket spending, Health expenditure tracking, System of Health Accounts, Tajikistan

## Abstract

This analysis examines trends in health spending in Tajikistan from 2000 to 2022 and situates them within the country’s key health financing reforms, with the aim of informing progress toward Universal Health Coverage (UHC) and identifying persistent gaps. By applying data from the WHO Global Health Expenditure Database (GHED), which is structured according to the System of Health Accounts (SHA 2011), and contextualizing results with national policy documents and relevant literature, the paper assesses both expenditure patterns and reform dynamics.

While government health spending has increased in absolute terms, it remains modest as a share of GDP and general government expenditure. Out of pocket payments have declined slightly as a share of current health spending, yet they still account for nearly two-thirds, posing barriers to equitable access and financial protection. Recent reform initiatives, such as pooling and strategic purchasing pilots, show potential to improve equity and efficiency; however, their long-term impact will depend on sustained implementation and systematic tracking of expenditures.

The findings underscore the importance of tracking expenditure in a systematic way to guide health reform. Institutionalizing the routine production of health accounts using SHA 2011 would improve transparency, strengthen allocative efficiency, and support more strategic resource allocation and informed policy dialogue. Ultimately, tracking health spending is not just a technical exercise, but a strategic tool to align financing with policy priorities and advance UHC.

## 1 Introduction

Health spending is not simply a matter of allocating financial resources, but rather a strategic investment that shapes health outcomes, equity, and economic development. Globally, robust evidence suggests that well-designed health financing policies are associated with stronger health systems and improved population health [1], [2], [3], [4], although causal effects depend on broader system and policy contexts.

The ability of countries to monitor how health resources are mobilized, pooled, and spent is increasingly seen as central to achieving Universal Health Coverage (UHC) and the Sustainable Development Goals [2], [5], [6]. As emphasized by the United Nations Inter-Agency Task Force on Financing for Development, tracking health expenditure is not merely about reporting numbers, it is a vital tool for informing policy decisions, enhancing transparency and accountability, and guiding health system reforms [6].

In Tajikistan, as in all other countries, the way resources are allocated has profound implications for equity and access, particularly in the context of ongoing economic and demographic transitions [7]. Over the past two decades, the country has evolved into a lower-middle-income economy, with its population growing from approximately 6.2 million in 2000 to around 10.1 million in 2022. Over this period, GDP per capita increased fourfold, underpinned by consistently positive growth averaging 5.2% annually during 2015–2022 and reaching 8% in 2022 [7], [8]. These gains have been accompanied by a notable decline in poverty levels and an increase in life expectancy at birth from 63.3 years to 71.3 years [7], [8]. Despite these gains, health outcomes continue to lag behind regional averages due to persistent service delivery gaps and limited access to essential medicines and diagnostics [9], [10], [11].

This article provides a comprehensive analysis of how health funds have been mobilized, allocated and spent in Tajikistan from 2000 to 2022, and examines the extent to which current patterns support progress toward UHC. Adequate and equitable use of resources is investigated by analyzing trends in current health expenditure (CHE), government health expenditure (GHE), and out of pocket (OOP) payments, as well as spending on primary health care (PHC), outpatient medicines, and disease-specific categories. The trend analysis highlights broad shifts in health spending but also reveals important data gaps, particularly regarding spending by disease area or service delivery type, such as preventive versus curative care, due to the absence of sufficiently detailed national expenditure data.

Addressing these limitations through regular health accounts production and improved reporting systems will be essential for tracking reform outcomes and progress toward equity and financial protection.

## 2 Materials and methods

The analysis was based on publicly available secondary data, complemented by selected national and international reports and contextual policy documents. Specifically, it drew on multiple sources commonly used in health financing research and monitoring including:

• The World Health Organization’s Global Health Expenditure Database (GHED)*,* which offers standardized, internationally comparable data compiled using the System of Health Accounts (SHA 2011) methodology [12], [13]. Countries report to GHED either through full health accounts exercises or by providing aggregated and estimated data consistent with the SHA framework. For Tajikistan, GHED data are based on aggregated administrative estimates rather than full health accounts, due to the absence of institutionalized national health accounts production.

We used data on CHE disaggregated by financing sources, including GHE, OOP spending and external funding from 2000 to 2022 [12]. Data on expenditure by health services and disease categories were included for selected years. While a complete time series was available for current expenditure and financing sources, some disaggregated data (e.g., by function or disease category) were available only for specific years. No imputation was applied; these results are presented as illustrative examples to demonstrate analytical potential and existing data limitations. Macroeconomic variables and deflators were obtained directly from GHED to ensure consistency across indicators.

All expenditure figures are expressed in constant 2022 US dollars, unless otherwise specified in the text. Using constant prices allows comparability over time by removing the effects of inflation. Given the focus on a single-country, time-series analysis, results are presented in USD rather than purchasing power parity, as the latter are more suitable for cross-country comparisons rather than trend analyses within a single national context.

GHED data used in this analysis were extracted on 28 June 2025 to ensure consistency and comparability across time [12].

• Supplemental studies*,* including national and external reports, policy reviews, and relevant literature, were used to provide additional contextual information and to help interpret observed spending patterns. Identification of these documents followed a targeted search strategy, drawing on institutional databases (e.g. WHO, World Bank), reference lists, and expert consultation. These qualitative materials provided insights into health financing reforms and helped to identify implementation gaps and systemic challenges.

The SHA 2011 methodological framework guided the identification of data gaps and interpretation of policy implications. Descriptive statistical analysis was used to examine expenditure trends, which were triangulated with policy reviews and supplementary evidence to assess the alignment of reforms with spending patterns. Documented evaluations were also consulted to identify promising practices and persistent misalignments between spending and national health priorities. Where relevant, the analysis accounted for the time lag between policy adoption and observable fiscal effects, distinguishing between earlier reforms with measurable impact during 2000–2022 and more recent initiatives whose effects extend beyond the study period.

## 3 Results

The descriptive trend analysis of health expenditure in Tajikistan, complemented by policy reviews and supplementary studies, provides an overview of significant shifts in spending patterns and their alignment with health financing reforms. We first describe overall CHE trends, then composition by financing sources (public, OOP, external), and finally functions (PHC, medicines) and disease categories.

### 3.1 Evolution of health financing reforms in Tajikistan

Following independence in 1991 and the end of the civil conflict in 1997, Tajikistan began transforming its centralized, hospital-oriented health system, long characterized by inefficiencies, excess capacity, and chronic underinvestment in PHC [14], [15]. In the immediate post-conflict years, health services were heavily supported by donors, and early reforms took the form of small-scale pilots in PHC financing and benefits design. These included experiments with capitation-based provider payments, performance-based financing (PBF), and the introduction of two complementary instruments defining entitlements and cost-sharing, the Basic Benefit Package (BBP) and Government Decree No. 600, which together established the framework for publicly funded services and the co-payment system and have remained the backbone of benefits regulation in Tajikistan [14].

These pilots were gradually consolidated under the National Health Strategy 2010–2020, which prioritized PHC strengthening, provider payment reforms, and financial protection. The current National Health Strategy 2021–2030 builds on this foundation, aiming to institutionalize reforms through resource pooling, strategic purchasing, and sustainable public investment. Recent initiatives, such as oblast-level pooling and pilot case-based payments in hospitals, reflect this direction [9], [14]. Yet, challenges remain, as fiscal space is constrained, OOP payments continue to dominate health financing, and systematic tracking of how funds are allocated across health priorities is still weak [7], [9], [14].

At the same time, limited national data granularity constrains detailed trend analysis and attribution of expenditure changes to specific reforms, underscoring the need to institutionalize health accounts production and strengthen routine financial reporting systems.

### 3.2 Trends in current health expenditure and financing composition

Tajikistan’s CHE has increased substantially over the past two decades. According to data from the WHO GHED, CHE as a share of GDP rose from 4.3% in 2000 to 7.6% in 2022. Over the same period, per capita CHE increased from USD 14 to USD 79, expressed in constant 2022 USD using WHO deflators, corresponding to an average annual growth rate of 8%.

A notable 30.6% increase in per capita CHE between 2019 and 2020 was driven by COVID-19-related emergency spending by the government and external donors and partners. While these constant-price figures capture real increases in health expenditure, improvements in access and outcomes cannot be directly attributed to higher spending alone, as they depend on how efficiently and equitably resources are allocated. Persistent challenges, including the growing burden of non-communicable diseases (NCDs), population ageing, and infrastructure gaps, highlight the need for better-targeted investment [7], [14].

The upward trend in CHE coincided with several health financing reforms, including the introduction of capitation-based payments in PHC and the development of formal benefit-defining instruments (BBP and Government Decree No. 600), which together structured entitlements and cost-sharing arrangements across different levels of care [14], [16], [17]. More recently, pilot reforms in oblast-level (regional) pooling and strategic purchasing contracts in selected rayons have aimed to improve equity and reduce fragmentation in financing [16]. Nonetheless, the structure of health financing remains skewed.

**Fig. 1:**
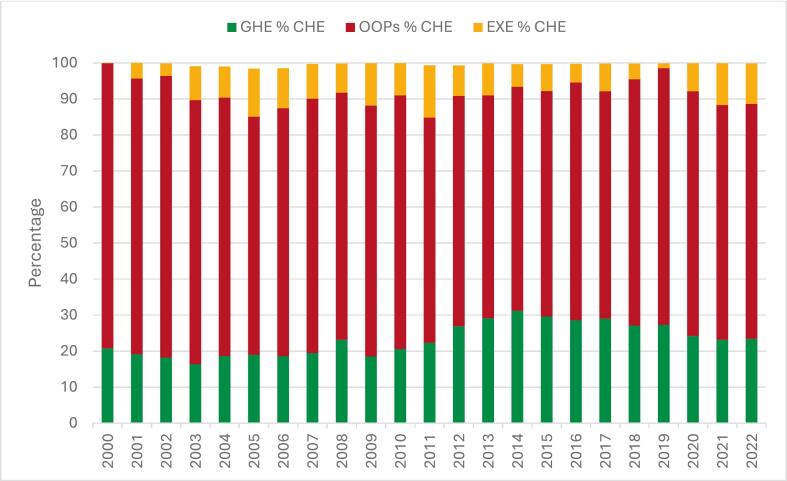
**Composition of CHE by GHE, OOP Spending, and EXE, 2000**–**2022.***.*

*Source: GHED, 2025*

Public spending accounts for only 20–30% of CHE, while OOP payments exceed 60% (Fig. 1). External funding from donors (EXE) remains a crucial source of health financing in Tajikistan, helping to fill some of these gaps. The share of external resources in CHE has fluctuated considerably, from almost 0% in 2000 to a peak of 14% in 2011, before stabilizing at around 11% in 2022 (Fig. 1). Despite this variability, support from external partners continues to play a critical role in sustaining service delivery and filling gaps in government financing [9].

### 3.3 Public financing trends and government prioritization

Government health expenditure in Tajikistan increased from USD 19 million to USD 187 million (in constant 2022 USD), representing nearly a tenfold rise over two decades. The average annual growth rate of about 9% reflects a steady upward trend in public investment in health, with the sharpest increase occurring between 2010 and 2020 when allocations more than tripled. Cumulatively, total current government health spending reached approximately USD 1.86 billion over the 20-year period, indicating sustained, though still modest, investment relative to the country’s health needs and fiscal capacity.

**Fig. 2:**
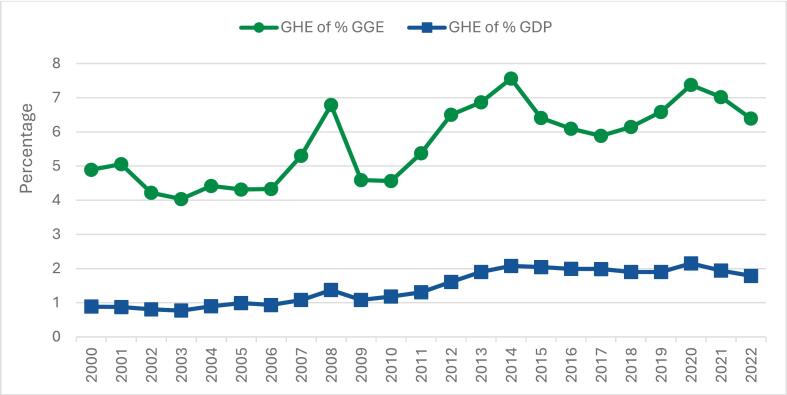
Priority given to health by the Tajik Government, 2000–2022. *.*

*Source: GHED, 2025*

GHE as a share of general government expenditure (GGE) peaked at 7.6% in 2014 and declined to 6.4% by 2022 (Fig. 2). This remains well below the Abuja Declaration target of allocating 15% of GGE to health, a benchmark often recommended by WHO to indicate strong government commitment to UHC [18]. Compared with other sectors such as education and infrastructure, health continues to receive a relatively modest share of the national budget [8], [9], [11].

Fig. 2 illustrates a similar pattern when GHE is expressed as a share of GDP, which increased from 0.8% in 2000 to 1.8% in 2022, with a temporary peak at 2.2% during the COVID-19 pandemic. After this peak, growth slowed in 2021–2022, suggesting renewed fiscal pressures or shifting government priorities. However, preliminary 2023 data indicate a possible recovery, with GHE rising to about USD 223 million.

### 3.4 Out-of-pocket spending – A persistent barrier to access and financial protection

Despite gradual increase in government health spending, OOP payments have remained the dominant source of health financing in Tajikistan. In distributional terms, evidence indicates that OOP payments are not evenly distributed but fall disproportionately on low-income and rural households, creating substantial financial barriers to accessing needed health services [10]. Analysis of CHE trends shows that OOP payments have consistently accounted for around two-thirds of CHE, although their share has gradually declined from 79% in the early 2000s to about 67% in recent years (Fig. 1). However, the trend has not been linear, and year-to-year fluctuations reflect policy instability, limited fiscal space, and persistent gaps in population coverage [10].

Our expenditure analysis suggests that provider payment reforms, such as the PBF mechanism introduced in 2013, may have contributed to easing household reliance on OOP payments during its implementation by improving access to PHC. While it is not possible to establish a direct causal effect based on this expenditure analysis, PBF can be considered a plausible contributing factor to the modest decline observed. The pilot was discontinued in 2023 due to fiscal constraints [9], [14], underscoring the challenge of sustaining such mechanisms and the continued vulnerability of households to high OOP spending.

### 3.5 Primary health care spending and the burden of outpatient medicines

One of the key dimensions of expenditure tracking under the SHA 2011 framework is the classification of health functions and providers, which allows assessment of how resources are distributed across levels of care [13]. This analysis focuses on PHC spending, one of the core indicators available in the GHED. PHC is a cornerstone of Tajikistan’s health system and a strategic priority under the National Health Strategy 2030, which emphasizes people-centred, community-based services as the foundation for UHC [14], [19]. Tracking PHC expenditure is therefore essential for assessing system orientation and equity, and for understanding whether public resources are directed toward front-line services where most health needs are met.

According to GHED data, PHC accounted for 44–47% of CHE between 2016 and 2019. However, public financing covered only 35–37% of this spending, meaning that out of total current PHC spending (in current USD) of USD 26 per capita in 2019, only USD 6 was funded by the government. Consequently, a substantial share of PHC spending continues to be financed through OOP payments, largely driven by household expenditure on outpatient medicines. Between 2016 and 2019, medicines accounted for 21–24% of CHE, while government spending on medicines represented only 4–6% of total GHE. Notably, medicines are only partially covered under the benefit-defining instruments, which limit financial protection and contribute to high OOP payments [10].

Due to data constraints, a comprehensive assessment of PHC expenditure trends in Tajikistan is not possible. GHED provides data only for 2016–2019, which restricts analysis of longer-term changes or links to key PHC-related reforms, such as capitation-based payments, the introduction of benefit-defining instruments, and PBF, as potential drivers of spending patterns.

While the SHA 2011 global definition supports international comparability, it may not fully reflect Tajikistan’s PHC delivery structure [20], [21]. A tailored national PHC classification is still under development, and the limited granularity of GHED data does not permit detailed analysis by provider or function. Establishing a country-specific PHC classification aligned with SHA 2011 principles would enhance policy relevance, enable monitoring of PHC prioritization, and support evidence-based resource allocation [21].

### 3.6 Disease-specific expenditure

Understanding how health spending is distributed across disease areas is essential for assessing whether resources are aligned with population health needs and national policy priorities. Tracking such expenditure is also a core element of analysis under the SHA 2011 methodology and provides valuable evidence for strategic planning and priority setting.

**Fig. 3:**
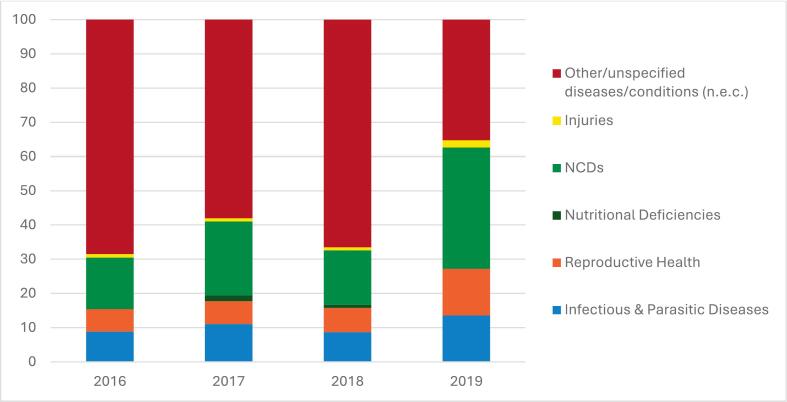
**Distribution of Health Expenditure by Disease Category, Tajikistan, 2016–2019.** .

Source: GHED (2025). Note: n.e.c − not elsewhere classified

In Tajikistan, disease-specific expenditure tracking remains weak due to data limitations. Between 2016 and 2019, a substantial share of CHE was categorized as “unspecified”, limiting the ability to assess alignment between spending patterns and the country’s disease burden or policy goals (Fig. 3). The available data nonetheless provides a useful snapshot of emerging trends. During this period, recorded expenditure on NCD increased reflecting their growing contribution to the national disease burden. NCDs now account for more than 80% of all deaths in Tajikistan, with cardiovascular diseases and diabetes among the leading causes [7], [14]. In contrast, expenditures on infectious and parasitic (communicable) diseases, reproductive health, and nutrition remain low, despite persistent challenges such as high maternal and child mortality and a relatively high burden of communicable diseases in remote regions [14].

## 4 Discussion

This analysis highlights Tajikistan’s mixed progress in health financing over the past two decades. Public spending has increased from a low base, several structural reforms have been piloted, and the health sector’s budget share has risen, indicating gradual prioritization. OOP payments as a share of CHE have declined modestly in recent years. Nevertheless, OOPs remain among the highest in the WHO European Region, continuing to constrain equitable access and financial protection [18].

### 4.1 Health-sector reforms and expenditure patterns

These results, when interpreted in the context of Tajikistan’s evolving reform landscape, highlight both achievements and persistent structural constraints. Since 2000, Tajikistan has pursued incremental reforms aimed at reorienting health financing toward greater equity and efficiency, consistent with the objectives of the two national health strategies for 2010–2020 and 2021–2030 [7], [9], [14].

As summarized in Box 1, these have included the introduction of capitation-based payments in PHC, the development of benefit-defining instruments (BBP and Government Decree No. 600) with specified entitlements and co-payment rules, pilots of PBF, and the launch of oblast-level pooling initiatives [9], [14], [16], [17]. These sequential reforms correspond with gradual shifts in financing patterns—particularly a rising public share and a modest reduction in OOP payments shown in Fig. 1, reflecting progress toward stated policy objectives and priorities outlined in the National Health Strategy 2021–2030.

Box 1 **Key selected health financing reforms in Tajikistan (2000–2023).**

Summary of selected major health financing reforms shaping Tajikistan’s health system between 2005 and 2023. *Source:* [14].

**Table:** 

**Period.**	**Reform / Instrument.**	**Main Focus and Description.**	**Status / Coverage.**
**2005 – present.**	**Capitation-based payments in PHC.**	Introduced to improve efficiency and equity in PHC resource allocation through per-capita financing of family medicine services. Piloted in Dangara and Varzob, expanded nationally now.	Implemented nationwide; refinements ongoing under the Strategy on Healthcare of the Population of the Republic of Tajikistan for the period up to 2030 (SHPRT 2030).
**2007 – 2023.**	**Basic Benefit Package (BBP)**	Defined publicly funded PHC and hospital services, including free family-doctor consultations and exemptions for vulnerable groups.	Piloted and revised multiple times; implementation ended May 2023 pending redesign.
**2008 – present.**	**Government Decree No. 600.**	Established the framework for entitlements, co-payment rules, and official price lists for inpatient and specialized outpatient care.	National regulation; under revision to align with SHPRT 2030.
**2013 – 2023.**	**Performance-Based Financing (PBF) pilots.**	Linked payments to results to improve PHC quality and coverage; implemented in 16 rayons under the *Health Services Improvement Project/World Bank*.	Completed pilots; not institutionalized nationally.
**2019 – present.**	**Oblast-level pooling and strategic purchasing pilots.**	Consolidated regional pooling and introduced contractual purchasing between payers and providers, starting in Sughd oblast.	Ongoing pilots under SHPRT 2030 .
**2010 – 2030.**	**National Health Strategies (2010–2020; 2021–2030)**	Provided overarching policy frameworks for financing reform, emphasizing PHC strengthening, equity, and financial protection.	SHPRT 2030 under active implementation.

However, many initiatives were implemented at pilot scale, were time-bound, or lacked integration into core budgetary processes, limiting their system-wide influence [9], [14]. Persistent misalignment between benefit design and actual resource flows, especially at the PHC level and for outpatient medicines, continues to weaken financial protection [10], [14], [17].

### 4.2 External constraints and fiscal environment

Reform progress in Tajikistan has unfolded within a constrained macro-fiscal environment. The share of general government expenditure devoted to health remains modest relative to population needs and international benchmarks, while dependence on external assistance continues [9], [14], [22]. Inflationary pressures, competing sectoral priorities, and limited fiscal space have collectively tempered growth in public spending, making recent reform gains incremental rather than transformative.

In regional perspective, Tajikistan continues to lag behind Kazakhstan, Kyrgyzstan, and Uzbekistan in per-capita public spending and faces higher OOP shares [12], [18]. Kyrgyzstan, for instance, introduced nationwide pooling and purchasing mechanisms earlier and, through institutionalized health-accounts reporting, achieved relatively higher public shares of CHE and greater financial protection [23]. These regional contrasts underscore both the structural limitations of Tajikistan’s fiscal capacity and the potential policy lessons from neighboring countries that have maintained higher and more predictable public investment in health.

A particularly persistent challenge concerns household spending on pharmaceuticals, which remains one of the main drivers of OOP payments. Limited coverage of outpatient medicines under benefit-defining instruments, coupled with weak procurement systems and price regulation, continues to erode financial protection [7], [9], [10], [16], [17].

Expanding public investment in essential medicines, improving procurement efficiency, and introducing rules-based purchasing mechanisms, particularly for outpatient drugs and PHC, would help reduce these pressures and strengthen equity in health financing.

### 4.3 Tracking expenditure and data systems

A central finding of this analysis is the limited systematic and disaggregated tracking of health expenditure for policy use. Tajikistan’s public financial management and treasury systems produce routine fiscal reports, but these do not provide the disease-, function-, or provider-level detail needed to assess how financing reforms affect spending patterns.

The GHED offers standardized, internationally comparable data, yet it remains highly aggregated and lacks the granularity required to monitor reform outcomes, particularly for PHC, medicines, and disease-specific spending [12], [13].

Although Tajikistan has previously produced full health accounts studies based on the SHA 2011, these were conducted only sporadically and, therefore, cannot support consistent time-series analysis linked to reforms.

The issue is not the absence of government financial reporting, but rather the lack of institutionalized, policy-relevant expenditure tracking, including OOP payments and external donors spending, produced on a regular cycle and linked to resource allocation planning and reform monitoring processes.

### 4.4 The role of institutionalized tracking of health expenditure

International experience shows that embedding a regular, policy-oriented health-expenditure tracking function within government systems can substantially improve the completeness, comparability, and policy relevance of spending data, thereby supporting budget negotiations, strategic purchasing, and health–finance dialogue [24], [25], [26], [27]. While routine tracking does not deliver real-time data, it provides consistent, methodologically coherent estimates by financing scheme, provider, function, and, where feasible, beneficiary group – information that can be integrated into health-sector planning and performance monitoring to assess reform outcomes [27], [28].

Kyrgyzstan offers a relevant example: through nationwide pooling and purchasing reforms and the institutionalization of full health-accounts reporting, the country created a robust evidence base that enabled continuous monitoring of reforms and informed dialogue between the health and finance authorities [23]. Thailand has long used National Health Accounts to underpin budget negotiations for its Universal Coverage Scheme and to guide purchasing and provider-payment reforms [29], [30]. Armenia similarly demonstrates how institutionalized expenditure tracking has been used to guide strategic purchasing decisions and adjustments to the benefit package, even under fiscal constraints [31], [32].

For Tajikistan, institutionalizing a periodic, policy-relevant health expenditure tracking mechanism and integrating its outputs into planning and policy review processes would strengthen transparency and allocative efficiency, complement existing Public Financial Management (PFM) reporting, and enable more systematic assessment of whether resources follow stated health priorities, particularly for PHC and essential outpatient medicines.

### 4.5 Policy implications

The analysis suggests several practical implications for strengthening Tajikistan’s health financing system. Establishing a regular, nationally owned health-expenditure tracking function would enable policy makers to identify inefficiencies, align resources with strategic priorities, and assess the distributional impact of financing reforms [27], [28].

Building on this, developing a country-specific framework for tracking PHC and medicines expenditure, building analytical capacity within the health and finance authorities, and ensuring the routine use of expenditure evidence in policy dialogue would help translate financial data into actionable decisions [20], [21]. In particular, institutionalizing full, routine health accounts would provide the detailed evidence base needed to monitor the effects of recent reforms, such as pooling and purchasing mechanisms, whose outcomes will only become visible over the longer term.

In practical terms, three near-term priorities follow: increase the public budget for health and rebalance spending toward PHC; reduce OOP payments by updating benefit-defining instruments to better cover essential outpatient medicines; and align provider-payment methods (capitation/case-based with budget caps) with purchasing objectives to improve efficiency. Collectively, these steps would enhance transparency, accountability, and the alignment of health spending with national UHC goals.

Given similar challenges, high OOP payments, partial outpatient medicines coverage, and intermittent health accounts production, across other Eastern European and Central Asian countries, these steps are regionally relevant and adaptable, with regional experience illustrating the value of institutionalized tracking and pooled purchasing [23], [29], [30], [31], [32].

## 5 Limitations

This analysis relies on secondary data from WHO GHED. As noted in the Methods section, for Tajikistan the submission is based on aggregated administrative estimates rather than full health accounts, which limits disaggregation by provider, function, or beneficiary group. The quality and estimation methods of some indicators, especially OOP spending, vary. GHED data are reported with a typical two-year time lag, affecting timeliness, although this can be reduced to around one year when institutionalized national health accounts are in place. These limitations underscore the importance of establishing regular, nationally produced health accounts to improve data quality, timeliness, and policy relevance for tracking reforms over time.

## 6 Conclusion

Tajikistan’s health financing has advanced through gradual reforms and increased public investment, reflecting a growing commitment to UHC. Yet progress remains uneven, constrained by several systemic gaps. Weak expenditure tracking and limited disaggregation hinder the ability to link spending to policy priorities; an incomplete classification of PHC services limits monitoring of front-line investments; the absence of systematic evaluation of pilot initiatives restricts learning and scale-up; and low public spending with fragmented financing continues to sustain high OOP payments and reliance on donors.

Addressing these challenges requires institutionalizing regular, policy-relevant health expenditure tracking, strengthening PHC expenditure classification, and embedding evaluation mechanisms into future reforms. Systematic tracking of health spending is foundational for transparency, evidence-based budgeting, and course-correcting reforms, a prerequisite for equitable, sustainable progress toward UHC in Tajikistan.

## Funding

This study was produced with funding assistance of the European Union. The contents of this publication are the sole responsibility of the authors, present their expert opinion, and can in no way be taken to reflect the views of the European Union.

## Declaration of competing interest

The authors declare that they have no known competing financial interests or personal relationships that could have appeared to influence the work reported in this paper.
